# Research progress on artificial intelligence technology-assisted diagnosis of thyroid diseases

**DOI:** 10.3389/fonc.2025.1536039

**Published:** 2025-02-20

**Authors:** Lina Yang, XinYuan Wang, Shixia Zhang, Kun Cao, Jianjun Yang

**Affiliations:** ^1^ Development Department of the Wisdom Hospital, Shandong Provincial Third Hospital, Jinan, China; ^2^ Information Department, Shandong First Rehabilitation Hospital, Linyi, China; ^3^ General Practice Medicine, Shandong Provincial Third Hospital, Jinan, China

**Keywords:** thyroid disease, machine learning, image recognition, thyroid ultrasound, thyroid pathological slices

## Abstract

With the rapid development of the “Internet + Medical” model, artificial intelligence technology has been widely used in the analysis of medical images. Among them, the technology of using deep learning algorithms to identify features of ultrasound and pathological images and realize intelligent diagnosis of diseases has entered the clinical verification stage. This study is based on the application research of artificial intelligence technology in medical diagnosis and reviews the early screening and diagnosis of thyroid diseases. The cure rate of thyroid disease is high in the early stage, but once it deteriorates into thyroid cancer, the risk of death and treatment costs of the patient increase. At present, the early diagnosis of the disease still depends on the examination equipment and the clinical experience of doctors, and there is a certain misdiagnosis rate. Based on the above background, it is particularly important to explore a technology that can achieve objective screening of thyroid lesions in the early stages. This paper provides a comprehensive review of recent research on the early diagnosis of thyroid diseases using artificial intelligence technology. It integrates the findings of multiple studies and that traditional machine learning algorithms are widely used as research objects. The convolutional neural network model has a high recognition accuracy for thyroid nodules and thyroid pathological cell lesions. U-Net network model can significantly improve the recognition accuracy of thyroid nodule ultrasound images when used as a segmentation algorithm. This article focuses on reviewing the intelligent recognition technology of thyroid ultrasound images and pathological sections, hoping to provide researchers with research ideas and help clinicians achieve intelligent early screening of thyroid cancer.

## 1 Introduction

The thyroid gland is a butterfly-shaped gland located in the front of the neck. Its main function is to secrete thyroid hormones. Thyroid hormones play a key role in regulating many physiological processes in the human body, including diabetes management, cardiovascular health, cognitive function, and immune system regulation. Therefore, maintaining normal thyroid hormone levels is essential to maintaining good health (1, 2). When thyroid hormone secretion is disordered, it can lead to abnormal thyroid function or abnormal thyroid structure. Thyroid dysfunction includes hyperthyroidism and hypothyroidism. Thyroid structural abnormalities mainly include thyroid nodules and thyroid cancer. Thyroid nodules refer to solid or cystic masses that appear inside the thyroid gland. Thyroid cancer is a malignant tumor that occurs in thyroid cells and is one of the most common malignant tumors in the endocrine system (3). The causes of thyroid cancer are complex. As a malignant tumor, tumor cells continue to grow and spread, leading to a decline in body function. During the diagnosis and treatment process, it may also cause emotional distress and psychological problems for patients. Studies have shown that cancer patients generally have a higher incidence of mood disorders such as depression and anxiety (4, 5).

Thyroid lesions often have no obvious symptoms in the early stages, but if not discovered and treated in time, they may gradually deteriorate into thyroid cancer, affecting the patient’s quality of life and even endangering their life. Therefore, although thyroid cancer has certain hazards, early detection, early diagnosis and early treatment can achieve better treatment results, reduce the surgery rate and mortality rate, improve the cure rate and reduce complications.

In recent years, significant changes in environmental factors, specifically manifested as heavy metal pollutants, persistent organic pollutants (POPs), and increased air pollution (6–8), have adversely affected the normal physiological functions of thyroid hormones. The incidence of thyroid cancer is increasing year by year globally, accounting for approximately 1% to 3% of all new malignant tumors worldwide (9). Currently, the methods for screening thyroid diseases include ultrasound, cell puncture, CT, MRI, etc (10–12). Ultrasound is a common non-invasive and painless examination method (13). Its disadvantage is that it is limited by the doctor’s experience and the size, shape, edge, internal echo and other characteristics of the nodule. Therefore, there is a certain misdiagnosis rate when evaluating the benign or malignant nature of thyroid nodules. Thyroid pathology is the gold standard for diagnosis and an important means of determining whether a thyroid nodule is benign or malignant and the type of thyroid tumor. However, pathology is invasive, expensive, and difficult for patients to accept. In order to achieve low-cost, high-accuracy early screening for thyroid disease, researchers have turned their attention to artificial intelligence technology.

The rapid advancement of artificial intelligence in image recognition technology has pushed auxiliary medical care to a highly mature and widely applied stage. In the field of image segmentation, deep learning image segmentation technology can automatically learn the features of images and achieve high-precision image segmentation by training deep neural. Xu (14) proposed an end-to-end FISH-based method (CACNET) for the recognition of genetically abnormal cells (CAC). The CACNET achieves cell nuclear segmentation by an improved Mask region-based convolutional neural network (R-CNN), and the accuracy of circulating CAC recognition using CACNET 94.06%. At the same time, they also developed a deep learning network (FISH-Net) based on 4-color FISH images for CACs, with an accuracy of more than 96% (15). Zhao (16) proposed a breast cancer ultrasound image segmentation method based on the U-Net framework combined with the residual block structure and attention, with a dic of up to 92.1%.

In the field of image classification, it mainly classifies and recognizes objects in images by training deep neural networks. This technology can process large-scale image data and quickly and accurately identify target objects in images. Its advantages include fast recognition speed, high accuracy, and the to handle images of different sizes and resolutions. In 2012, the deep convolutional neural network achieved a significant breakthrough in the ImageNet competition, showing excellent performance of 37.5% top-1 error rate and 17.0% top-5 error rate (17). In addition, Levy (18) proposed an innovative deep convolutional neural network model that cleverly used deep transfer learning technology to successfully achieve high-precision classification of benign and malignant breast tumors with an accuracy rate of up to 92.4%.

Wang (19) developed a mitosis detection method (FMDet) based on breast tissue histopathological images to capture the appearance changes mitotic cells. To achieve more robust feature extraction, the feature extractor was constructed by integrating a channel-level multi-scale attention mechanism into the fully convolutional network structure. The FMDet algorithm has won the first place in the MIDOG 2021 challenge, achieving an accuracy of 74.4%. In 2022, Su (20) used the gene expression data of TCGA to screen characteristic genes by combining WGCNA Lasso algorithms, and used machine learning models to achieve the diagnosis and staging of colorectal cancer. Wang (21) proposed a supervised learning (SSL) scheme of deep learning (DL) framework to address the challenge of high-precision classification seven pulmonary tumor growth patterns in whole slide images (WSIs). This series of technological innovations has undoubtedly injected strong impetus into the field of image segmentation and recognition, and has greatly promoted the application and development of artificial intelligence in early screening of thyroid diseases.

This article analyzes the application of artificial intelligence technology in the early diagnosis of thyroid diseases by comparing a large number of studies, summarizes the current application status of artificial intelligence technology in the early diagnosis of thyroid diseases, and studies the intelligent recognition technology of thyroid ultrasound images and pathological sections respectively. The aim is to explore a technology that can achieve objective screening of thyroid lesions in the early stages. Based on literature research, we explored the application of machine learning and deep learning in thyroid auxiliary diagnosis. We find that for small sample data, SVM and semi-supervised neural networks in deep learning perform better. U-Net has become the benchmark for most image segmentation tasks, with an accuracy of more than 93%, thanks to its encoder-decoder architecture. Artificial intelligence technology enables auxiliary examination for early screening of thyroid diseases, improving the early cure rate and survival rate of patients, and enhancing the accuracy and of doctors’ diagnosis. This study also prospects the future trends of artificial intelligence in the field of thyroid disease research, and constructs a set of artificial intelligence system for the whole process. The development of artificial intelligence in thyroid disease research is no longer limited to thyroid pathology or thyroid ultrasound, but has created an artificial intelligence that integrates thyroid images and clinical data of thyroid cancer, which is used to determine the diagnosis of thyroid cancer and can also accurately predict the postoperative survival period of thyroid cancer patients.

## 2 Methods

**Figure 1:**
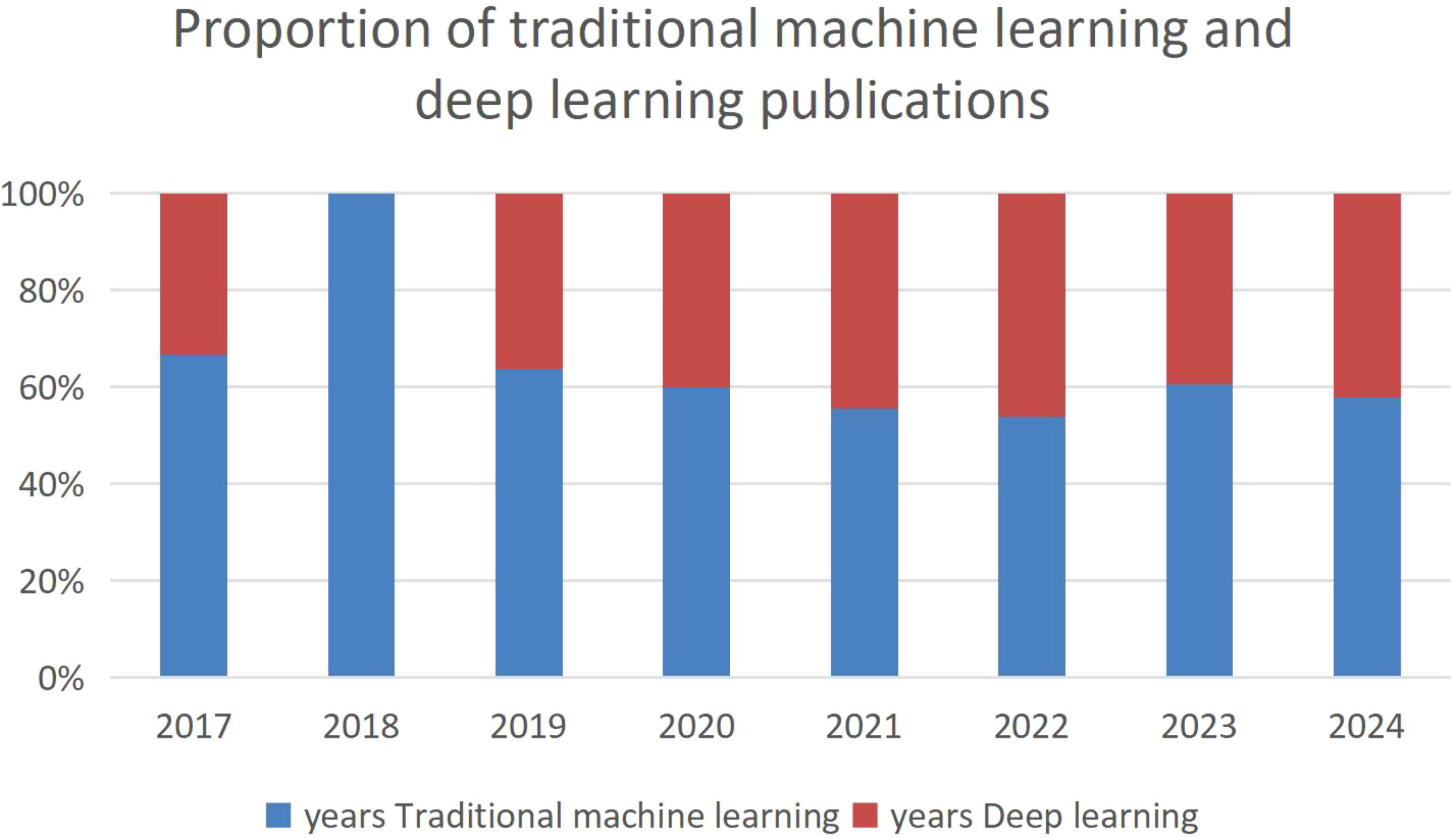
Proportion of traditional machine learning and deep learning publications.

The PubMed database was accessed by computer for retrieval, using “thyroid ultrasound”, “thyroid cytopathology” and “machine learning” as search terms. **Figure 1** shows the number of publications in the field of thyroid in the past decade. A total of 75 articles were selected for analysis. According to the inclusion and exclusion criteria, 50 articles were finally determined for research and analysis. The inclusion criteria for this review were: (1) Machine learning and deep learning algorithms, such as U-net, K nearest neighbor classification, random forest, support vector machine and artificial neural network. (2) The accuracy of early diagnosis of thyroid disease area under the receiver operating characteristic curve. (3) The time selection is the literature published in 2014 and later in the past 10 years. (4) Except for the GLAS and RITE public datasets, most of them are self-built datasets, which reviewed the data of thyroid patients for years, including thyroid ultrasound images and thyroid pathological slices. The following summary measures were used: machine learning method, sample size, performance measure, and important features. In the early diagnosis of thyroid diseases, the successful application of artificial intelligence technology mainly focuses on two core areas: traditional machine learning methods and deep learning methods.

(1) Traditional Machine Learning: The goal is to train algorithms by analyzing data so that computers can automatically identify and make appropriate decisions (22). Machine learning can be divided into two main types of learning methods: supervised learning and unsupervised learning, which are widely used in many fields such as medical diagnosis, image recognition technology, and sentiment analysis (23). The significant progress made by machine learning in the field of medical image analysis has provided strong technical support for the early screening of thyroid diseases. For example, a study used a dataset from the UCI machine learning library to train a multi-class SVM classifier to classify thyroid diseases (24). The Thy-Wise model uses a random forest algorithm to classify thyroid nodules, showing high accuracy and specificity while reducing the rate of unnecessary biopsies (25).

(2) Deep Learning: Compared with traditional machine learning methods, deep learning has powerful learning capabilities and can better utilize data sets for feature extraction (26). The key technologies of deep learning include convolutional neural network (CNN), recurrent neural network (RNN) and U-Net (27). Deep learning technology has shown great potential and advantages in the classification, detection and segmentation of medical images. For example, the application of U-Net model in biomedical image segmentation (28) and the success of deep residual network in image recognition (29) have demonstrated the effectiveness of deep learning technology in processing complex medical image data.

## 3 Results

### 3.1 Thyroid ultrasound image recognition technology

Thyroid ultrasound diagnosis uses the principle of ultrasonic wave propagation and reflection in human tissues. It transmits ultrasonic waves to thyroid tissues through high-frequency probes, collects the reflected echo signals, and forms ultrasonic images of the thyroid gland. These images can clearly show the size, shape, structure and blood flow of the thyroid gland, providing doctors with rich diagnostic information. Due to its significant advantages of fast imaging, non-invasiveness and no radiation, it has become a widely used and trusted examination method (30–32). Although ultrasound technology has many significant advantages, it also faces some inherent limitations. First, it is unavoidable interference noise and possible artifacts. Second, the shape of thyroid nodules is complex and changeable, blurred, and discontinuous. The boundary characteristics. Third, it is limited by the subjective experience of doctors. These problems have brought certain challenges to accurate diagnosis (33, 34). Therefore, exploring the application of artificial intelligence technology to assist in the diagnosis of thyroid ultrasound has become a research hotspot. **Table 1** shows some specific achievements artificial intelligence in the recognition of thyroid ultrasound images.

**Table 1:** The main results of machine learning algorithms in the study of thyroid nodule ultrasound images.

Published year	Reference	Type of DL	Main Performance	Data	Conclusion
2017	Raghavendra et al. (35)	SVM	ACC: 97.5%, AUC: 94%	242 ultrasound images	spatial gray-level dependence features (SGLDF) and fractal texture.
2017	Ma et al. (36)	CNN	ACC: 91.5%	22123 ultrasound images	A multi-view strategy is used to improve the performance of the CNN based model.
2019	Nguyen et al. (37)	DCNN	Accuracy: 90.88%	237 nodules	cascade classifier
2019	Fu et al. (38)	RF,SVM	RF AUC: 95.4%, SVM AUC: 95.4%	1179 nodules(including 501 benign and 678 malignant)	The performance of RF and SVM is superior to other methods.
2020	Shin et al. (39)	SVM	ACC: 69.0%, Specificity: 79.4%, Sensitivity: 41.7%	348 nodules	GLCM, GLRLM, Gabor, and Haar wavelet
2021	Vadhiraj et al. (40)	MIL	ACC: 96%	99 patients (33 benign, 66 malignant)	GLCM
2021	Peng et al. (41)	ThyNet	AUR: 92.2%	18049 ultrasound images	The proportion of missed malignant thyroid nodules has decreased.
2022	Zhou et al. (42)	MSA-UNet	ACC: 94.6%, Dic: 84.6%	1083 patients	Atrous Spatial Pyramid Pooling.
2023	Li et al. (43)	WSDAC	Dic: 87%	350 ultrasound images	Models can reduce the workload of labeling datasets.
2024	Chen et al. (44)	CNN	CNN AUC: 91%, Inception-ResNet AUC: 94%	11201 ultrasound images	The article conducted substantial, non-substantial, and benign malignant classification studies on ultrasound images. Inception-ResNet, due to the expertise of a senior doctor.
2024	Ma et al. (45)	KNN	ACC: 86.7%	508 ultrasound images	The study considered the impact of different distance weights, k-values, and distance metrics on the classification results.

#### 3.1.1 Traditional machine learning

In previous studies, ultrasound thyroid nodule segmentation methods can be roughly divided into four categories: shape and contour-based (46), region-based (47), machine learning-based (48), and hybrid methods (49).

At the beginning of the introduction of artificial intelligence technology in the medical field, researchers mainly relied on traditional machine learning algorithms. Therefore, the traditional machine algorithm was applied to the diagnosis of thyroid ultrasound images, aiming at improving the diagnostic speed and accuracy of benign and malignant nodules. In 2017, Raghavendra (35) designed a computer-aided diagnosis system (CAD) for the diagnosis of nodules. The system identifies the lesion area by integrating spatial gray-level dependence features (SGLDF) and fractal texture. This feature fusion-based approach achieved an accuracy of 97.5% and an AUC value of 94% for the support SVM using only two features, which is about 3.5% higher than the performance of the SVM proposed by Acharya et al. (50) How to use the right features to improve classification performance has always been a challenge.

Shin I (39) developed an artificial neural network (ANN) based on SVM for the classification model of thyroid tumors in 2020, using 348 preoperative ultrasound images of thyroid nodules as the dataset, and selected 10 important features as the feature input of the model. Then, the effect of the model was compared with the results of manual diagnosis by experienced radiologists. The results showed that the sensitivity, specificity and accuracy of the model were 32.3%, 90.1% and 74.%, respectively, while the sensitivity, specificity and accuracy of the diagnosis by general physicians were 24.0%, 84.0% and 648%. It was proved that the classifier model of machine learning may be helpful in the diagnosis of thyroid cancer.

In 2021, Vadhirajt (40) developed a computer-aided diagnosis system integrating multiple instance learning (MIL) to classify benign and malignant thyroid ultrasound images. Seven ultrasound image features were extracted using the gray-level co-occurrence matrix (GLCM) with an accuracy of 96%. Ma (45) proposed an improved KNN algorithm for automatic classification of thyroid nodules. The paper not only considered the number of class labels of various data categories in KNNs, but also considered the corresponding weights, using the Minkowski distance measurement. Using 508 thyroid nodule hyper images, the improved KNN accuracy was 86.7%. Through summarizing and analyzing the previous studies, we find that different feature selection will have a certain impact on the accuracy of the model.

At the same time, in order to evaluate which algorithm in linear and nonlinear machine learning is better for the benign and malignant classification diagnosis of thyroid nodules, Fu (38) used three linear and five nonlinear machine learning algorithms to evaluate 1039 patients with a total of 1179 nodules. Experimental results have shown that the AUC of machine learning models is higher than that of experienced radiologists. Among them, the AUC of RF and SVM methods in nonlinear machine learning is the highest, both at 95.4%, while the AUC of experienced doctors is only about 83%.

At present, a large number of computer-aided diagnosis systems based on traditional machine learning rely mainly on a variety of texture features and machine learning algorithms differentiating the benign and malignant nature of thyroid nodules, and their accuracy is about 3% higher than that of general doctors. In order to further improve the classification accuracy, the researchers adopted a variety of optimization methods, such as GLCM, SGLDF, to fine-tune the input features and parameters of the machine learning models, making these models show applicability in thyroid diagnosis.

#### 3.1.2 Deep learning

With the continuous advancement of artificial intelligence technology, the application of deep learning in the medical field has become the focus of research. In 2017, Ma (36) first attempted to use a CNN-based model for thyroid nodule segmentation and compared this method with six methods including GA-VBAC, JET, DRLS, SNDRLS, SVM-based method and RBFNN-based method. The study used a total of 22123 thyroid ultrasound images from three hospitals as the dataset. The results show that our proposed CNN-based model has a good performance in the segmentation of thyroid nodules with an accuracy of 91.5%. Peng (41) developed a deep learning model based on ThyNet to distinguish benign and malignant thyroid nodules, and the results showed that the AUC was 92.2%, and the proportion of missed malignant thyroid nodules decreased from 18.9% to 17.0%, reducing fine needle aspiration examinations. In 2024, Chen (44) proposed a convolutional neural network (CNN) model using 11201 images for training, validation and testing. Experiments have shown that the AUC of the model in the classification of benign and malignant thyroid nodules is higher than 91%, among which Inception-ResNet has the highest AUC of 94%, and the performance of the model is better than that of senior physicians.

In artificial intelligence applications, feature selection is key to improving model accuracy. In 2019, Nguyen (37) developed a method for extracting features from thyroid images, using a cascade classifier architecture to improve performance of computer-aided diagnosis systems for thyroid nodule classification. This method combined handcrafted standards and deep learning, achieving a classification accuracy of 90.8%. Gong (51) designed a new multi-task learning framework to simultaneously learn nodule size, glandular location, and nodule position, and proposed an adaptive glandular region feature enhancement module to fully utilize thyroid prior knowledge and use the prior to guide the feature enhancement network to accurately segment thyroid nodules. Different radiomic features were extracted from ultrasound images, including intensity, shape, and texture feature sets.

Although the popularity of deep learning has significantly improved the accuracy of image segmentation, problems with datasets, especially the lack of precisely annotated datasets, can still affect prediction accuracy of models. However, such data is often difficult to obtain in the field of medical image analysis. To solve this problem, Wang (52) proposed an attention-based semi-supervised neural network for thyroid nodule segmentation. The network can complete the thyroid ultrasound image segmentation task using a small amount of fully annotated data and a large amount of weakly annotated data. The article proposes two attention modules, which realize the inhibition or activation of bottom-up and top-down feature channels and image areas through a trainable feed-forward structure, thereby improving network performance. The Jaccard similarity coefficient of the semi-supervised neural network based on attention is 74.91%, which is 4.9% higher than that of the semi-supervised model based on VGG. The accuracy of benign and malignant thyroid tumor classification was improved from 91.67% to 95.00%, which proved that model had good generalization ability.

Li (43) proposed a weakly supervised deep active contour model for thyroid nodule segmentation, aiming to achieve accurate target segmentation with a small amount of annotation information. The experiment designed three modules: a weakly supervised learning framework, a deep active contour model, and auxiliary edge attention, which can reduce the annotation cost while maintaining a certain segmentation accuracy. The dic value of the model is 87%, which can reduce the workload of dataset annotation.

With the widespread application of deep learning, the U-net algorithm was proposed. U-Net is a convolutional neural network (CNN) structure widely used in deep learning, mainly for image segmentation tasks (53, 54). Ding (55) mainly explored the automatic segmentation technology of thyroid ultrasound images based on U-net. The model embedded an improved residual unit in the jump connection between the encoder and decoder paths and introduced an attention gate mechanism to enhance the weights of feature maps obtained from shallow and deep layers. Experimental results show that the proposed method outperforms other U-shaped models.

In 2020, Zhang (56) proposed two network structures, Cascade U-Net and CH-UNet, for the segmentation and classification of thyroid nodules. Cascade U-Net gradually refines the segmentation results and improves the segmentation accuracy by cascading multiple U-Net modules. CH-UNet combines dilated convolution and hybrid attention mechanism to enhance feature extraction and classification capabilities. Compared with the U-Net proposed by RONNEBERGER (55), the dice of Cascade U-Net in the task of thyroidodule segmentation increased by 2.9%. The dice of the U-Net method by RONNEBERGER (57) was only 80.2%, which fully validated effectiveness of the Cascade U-Net in the segmentation and even classification tasks of thyroid nodules.

In order to accurately detect malignant nodules that are not obvious and have confused boundaries in ultrasound images, and to avoid confusion between tissue and malignant thyroid nod during diagnosis, Yang (58) proposed a deep learning-based thyroid malignant nodule segmentation method of DMU-Net. The method uses the image context information in the U-shaped subnetwork to accurately locate the malignant nodule region, and then captures the fine details of theodule edges in the inverse U-shaped subnetwork. The combination of U-shaped subnetwork and inverse U-shaped subnetwork and the strategy of mutual learning make the dic of DMU-Net on the-built dataset 82.77%, which is 25.86% higher than that of the traditional U-Net network. The research proves that DMU-Net can accurately locate the malignant nodule area by extracting image context information in the U-shaped subnetwork, extract more lesion area features, and help radiologists diagnose thyroid diseases.

In 2022, Zhou (42) proposed an MSA-UNet model with a multi-scale self-attention mechanism for thyroid nodule segmentation. Depth wise separable convolution is used in the Atrous Spatial Pyramid Pooling (ASPP) module, and then in the decoder part, adjacent information of different scales is fused through the channel attention mechanism, allowing the model to learn more important features. The experimental results show that the accuracy of this method is 94.6%, which provides a new research idea for the early detection of thyroid nod. Comparison of accuracy of different U-Net algorithms, as shown in **Table 2**.

**Table 2:** Comparison of U-Net methods.

Reference	Methods	Recall	Accuracy	Dice
Ronneberger (57)	U-Net	86.1	93.2	80.2
Badrinarayanan (59)	SegNet	88.5	94	81.2
Zhou (60)	UNET++	85.9	93.8	80.8
Zhang et al. (56)	Cascade U-Net	86.6	94.3	83.1
Zhou et al. (42)	MSA-UNet	87	94.6	84.6

Currently, the research focus of thyroid ultrasound images is mainly on the segmentation and classification tasks of thyroid nodules, but the potential intrinsic connection and mutual influence between nodule characteristics and classification results are often ignored. Thyroid nodule segmentation and classification in ultrasound images are two fundamental but challenging tasks in computer-aided diagnosis of thyroid diseases. Since these two tasks are intrinsically related and share some common features, it is a promising direction to jointly solve these two problems using multi-task learning. However, previous studies have only demonstrated inconsistent predictions between these related tasks. In order to further exploit the effectiveness of the proposed task consistency learning, Kang (61) designed a framework based on multi-task learning (MS-MTL) to improve the performance of thyroid segmentation and classification by improving the consistency between tasks. The first stage of the network performs binary segmentation and classification simultaneously, and the second stage of the network learns multi-class segmentation. The article verifies the feasibility of improving thyroid nodule segmentation and classification performance through multi-task learning and inter-task consistency loss.

The application of deep learning in thyroid ultrasound images has broad significance and value. Various models have been applied to the processing of thyroid ultrasound images, including convolutional neural networks (CNN), U-net etc. By training a large amount of data, these models can learn the key features in ultrasound images for the classification and identification of nodules, thereby reducing misdiagnosis and missed diagnosis caused by human factors and helping to improve the early diagnosis rate. The application of artificial intelligence technology to assist in the early screening of thyroid diseases is not only limited to the diagnosis of thyroid ultrasound pictures, but also shows significant results in the recognition of thyroid pathology icons.

### 3.2 Thyroid pathology section recognition technology

**Figure 2:**
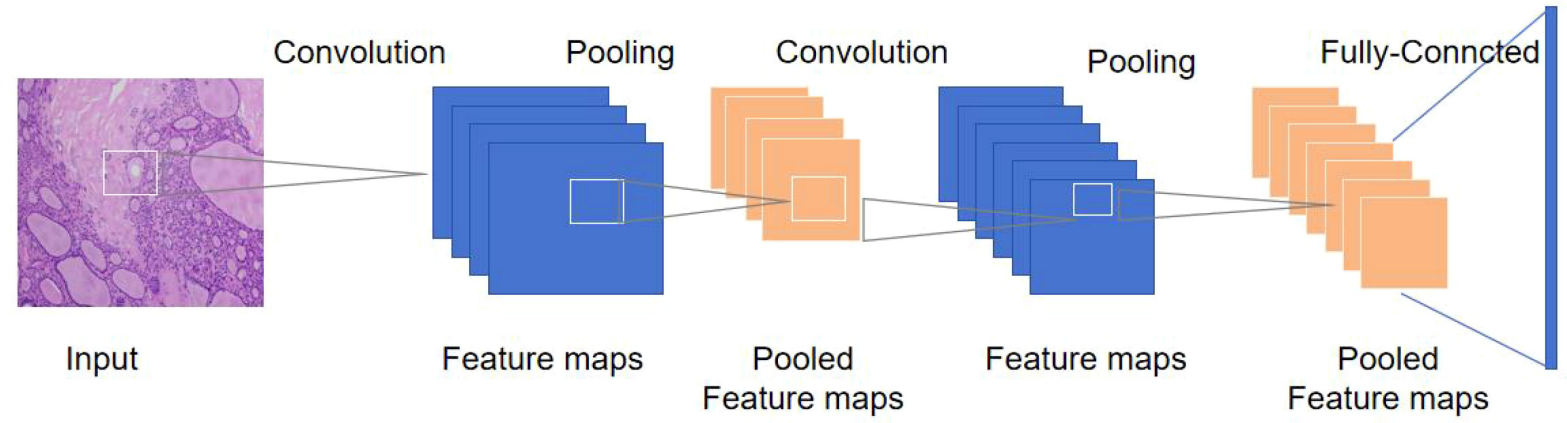
Convolutional neural network processing model for thyroid pathological images.

Thyroid pathology examination is a common diagnostic procedure and an important part of the evaluation of thyroid nodules, but there is significant variability in the assessment thyroid cytology specimens by different pathologists and institutions. The sensitivity reported in the literature ranges from 68% to 98%, and the specificity ranges from 56% to 100%. In this case, the use of machine learning can improve accuracy and help standardize the diagnosis of thyroid pathological specimens (62). The process of processing pathological images using convolutional neural networks is shown in **Figure 2**.

One of the earliest studies on thyroid pathology was conducted by Karakitsos (63), who investigated the ability of a learning vector quantization (LVQ) neural network to distinguish benign from malignant thyroid lesions. The model was trained by measuring 25 features such as size, shape, and texture of approximately 100 nuclei in each case. The results of the study show that the LVQ neural network can distinguish benign from malignant lesions very well, with an accuracy of 90.6%.

In 2011 study also investigated the application of learning vector quantization (LVQ) neural networks in differentiating benign from malignant thyroid lesions using 335 fluid-based cytology, fine needle aspiration (FNA), and Papanicolaou stain specimens. Features extracted by a custom image analysis system were first used to classify each nucleus using an LVQ neural network, and then a second LVQ neural network was used to classify individual lesions. The system was able to distinguish between benign and malignant nuclei and lesions at both the cellular and patient levels (64). Lee (65) developed a machine learning algorithm (MLA) that can classify human thyroid cell clusters by utilizing Papanicolaou staining and intrinsic refractive index (RI) as relevant imaging contrast agents and evaluated the impact of this combination on diagnostic performance. The accuracy of the MLA classifier for 1535 thyroid cell clusters from 124 patients using color images, RI images, and both was 98.0%, 98.0%, and 100%, respectively. The importance of this study lies in the fact that it compares a variety of different diagnostic techniques to improve the accuracy and efficiency of thyroid cancer diagnosis, with MLA classifier achieving the highest accuracy.

Artificial intelligence technology not only achieves precise classification and recognition functions in the processing of thyroid pathological images, but also shows strong prediction capabilities. Improving the of malignant tumor prediction can reduce the incidence of unnecessary surgery. Elliott (66) created a machine learning algorithm (MLA) based on two CNNs to identify follicular cells and predict the malign of the final pathology. The AUC of the model reached 93.2%, which is equivalent to the AUC of 93.1% diagnosed by cell pathologists, demonstrating the effectiveness of the algorithm. Wang (67) developed a prediction system for benign and malignant medullary thyroid cancer and goiter based on SVM and RF algorithms. For the classification of PTC and nodular goiter (NG), the SVM and RF algorithms performed equally well, with 94.2% and 94.4% consistency between the prediction and pathological diagnosis. The system can shorten the diagnosis time of doctors, making the diagnosis time of each sample only 10 minutes, which is very promising for the diagnosis papillary thyroid carcinoma during surgery. This method can also correctly predict the malignancy of a medullary thyroid carcinoma and a follicular thyroid adenoma.

Due to the combined effect of genetic variants, environmental exposure, and immune genetic risk (68, 69), new types of thyroid tumors, as” non-invasive follicular thyroid neoplasm”(NIFTP), have emerged, which has complicated the cytology of thyroid cells, and a lot data have been classified into the category of uncertainty (70).

Hirokawa (71) proposed an artificial intelligence image classification system of EfficientNetV2-L, which proved the efficiency and of artificial intelligence image classification system in identifying thyroid lesions. The research team used 148,395 thyroid pathology smear images from 393 thyroid nodules as the dataset. The researchers reported that the AUC of EfficientNetV2-L exceeded 95%. However, the AUC for poorly differentiated thyroid cancer was only 49%, showing significantly worse performance.

In another study, Yao (72) proposed a digital image analysis method based on feature engineering and supervised machine learning. They focused on cases of poorly differentiated thyroid cancer that were later diagnosed as benign or follicular adenoma in his tissue sections. The method was applied to 40 thyroid pathological slices with high and low power microscopy, and the AUC for the low power image model was 5%, and the AUC for the high power image model was 74%. This method performs better than cellular pathologists in classifying atypical follicular lesions.

The application of artificial intelligence in the field of thyroid pathology image analysis not only significantly enhances the accuracy and timeliness of diagnosis (73), but also relies on its deep learning and image processing technology to realize the analysis of pathological images such as follicular cell morphology and arrangement. Accurate identification of subtle features such as pattern and abnormal proliferation. These key features are of irreplaceable importance for accurately distinguishing benign and malignant thyroid nodules. Compared with traditional manual diagnostic methods, the integration of artificial intelligence has greatly promoted the early detection, accurate diagnosis and timely treatment of thyroid diseases, bringing patients a higher survival rate and better quality of life.

## 4 Discussion

In recent years, the research, development and application of artificial intelligence in the field of thyroid diagnosis have achieved significant leaps, providing new horizons and broad possibilities for optimizing the efficiency and accuracy of future diagnostic processes. Especially in the early diagnosis of thyroid cancer, artificial intelligence technology can automatically identify and evaluate complex medical images through machine learning algorithms, thereby improving the accuracy and efficiency of diagnosis.

In the application of thyroid ultrasound images, AI technology has been shown to effectively assist radiologists in the diagnosis of thyroid nodules. For example, one study showed that the performance of an AI system in the diagnosis of thyroid nodules was comparable to that of fine needle aspiration cytology (74). In addition, AI technology also showed high accuracy and efficiency in distinguishing benign from malignant thyroid nodules (75). Based on the previous research, we find that the research methods of thyroid ultrasound images mainly focus on traditional machine learning and deep learning. In traditional machine learning, SVM and RF have high accuracy in thyroid nodule classification due to their superior binary classification performance.

The core concept of SVM lies in the strategy of structural risk minimization, aiming to determine the optimal complexity of the model a limited dataset, thereby enhancing the model’s general prediction capability. The model parameters of SVM only depend on the support vectors, which are the data points closest to the decision boundary, and have no direct connection with other points. This means that even with a small number of samples, as long as these support vectors can fully reflect the overall distribution characteristics of the data, SVM can construct an efficient and accurate classification model. Therefore, SVM is particularly suitable for dealing with thyroid datasets with a small sample size.

Compared with machine learning, deep learning has strong learning ability and efficient feature expression ability, which can automatically learn and extract high-level features in images and can more comprehensively capture the details and context information of images, thus improving the accuracy of classification. The deep convolutional neural network (DCNN) model proposed by Krizhevsky (76) achieved breakthrough results in the ImageNet image classification. Therefore, the current research focuses on the classification of thyroid ultrasound and pathological images using deep learning.

Compared with traditional segmentation techniques, the segmentation method based on deep learning does not rely on hand-designed features, and the convolutional neural network (CNN) has shown excellent adaptability in the field of medical image segmentation by virtue of its image hierarchical feature representation capability. ROMÁN (77) reviewed a large number of deep learning-based medical image segmentation methods, among which U-Net is the most typical. The core idea of U-Net is to adopt a symmetric encoder-decoder architecture, which enables deep feature extraction and precise pixel-level segmentation of the input. Liu (78) proposed an automated segmentation algorithm for brain gliomas based on a multi-U-Net network(MU-Net), and conducted experiments on the BRATS2020 dataset. The results show that the Dice coefficients of the MU-Net algorithm for the complete tumor, tumor core, and enhanced tumor are 86.7%, 77.76%, and 76.21%, respectively, which are 2.6%, 2.55%, and 2.41% higher than those of the benchmark model, indicating better segmentation results. The application of these technologies can not only help radiologists diagnose thyroid diseases more accurately and improve diagnostic efficiency, but also reduce their workload.

AI technology also shows great potential in the application of thyroid pathology images. For example, AI technology has been used in cytological analysis of thyroid fine needle aspiration biopsy to distinguish papillary carcinoma from other types of thyroid cancer (79). A hybrid framework combining artificial intelligence was proposed in the study (80), which not only weighted the Thyroid Imaging Reporting and Data System (TIRADS) features, but also used the malignancy score predicted by the convolutional neural network (CNN) to classify and diagnose the malignancy of the nodules.

In summary, artificial intelligence technology has strong clinical significance and application prospects in the application of thyroid ultrasound images and thyroid pathological images. Not only has it improved the accuracy and efficiency of diagnosis, assisted doctors in decision-making, reduced the rate of misdiagnosis, but it can also the allocation of medical resources, reduce unnecessary surgeries and other invasive treatments through artificial intelligence-assisted diagnosis, and reduce the economic burden and pain of patients.

With the continuous advancement of technology and the deepening of clinical applications, artificial intelligence technology has played an increasingly important role in the early diagnosis of thyroid diseases, but the prediction of the postoperative life cycle of thyroid cancer patients is equally important for doctors and patients. This study (81) used artificial neural networks (ANN) to predict the 1-year, 3-year, and 5-year survival of thyroid cancer patients, with accuracy rates of 92.9%, 85.1%, and 86.8%, respectively. Based on our research results, artificial neural networks can effectively represent a survival prediction method for thyroid cancer patients. Liu (9) developed six machine learning models (SVM, XGBoost, LR, DT, RF and KNN) based on the SEER database to predict lung metastasis of thyroid cancer. Although the accuracy of the model is above 90%, prospective studies are still needed to further verify the practicality of the model. And because the genes of thyroid cancer patients may undergo mutation, gene mutation increases the complexity of the data, and the model may have difficulty accurately distinguishing different of diseases. On the other hand, gene mutation may have a complex interaction with other biomarkers or clinical information, which may make a single classification algorithm fail to capture the information accurately (82), and all these will lead to a bias in the accuracy of the algorithm model.

In the future, we will focus on optimizing the cutting-edge exploration of machine learning algorithm models, integrating patient pathological information, radiology and clinical information, create a more powerful algorithm, aiming to build a set of artificial intelligence system for the whole process. The system will have the ability to deeply analyze massive clinical records and molecular biology data to accurately predict the postoperative survival of thyroid cancer patients, thereby assisting doctors in tailoring more precise treatment strategies for each patient, thereby significantly improving late-stage Prognosis and quality of life in patients with thyroid cancer.

## 5 Conclusions

This paper reviews the latest application progress of artificial intelligence technology in the field of medical diagnosis, focusing on its potential in the early screening and diagnosis of thyroid. The research hotspot has developed from the initial traditional machine learning to deep learning algorithms, and U-Net has also become the benchmark for most medical image segmentation with the encoder-decoder architecture. Through the previous research, it aims to assist clinicians in achieving intelligent and efficient early identification of thyroid cancer, thereby improving the accuracy of early diagnosis for patients enhancing the efficiency of doctors. Moreover, the article also prospects the future trend of artificial intelligence in the field of thyroid disease research, not only limited to thyroid pathology or thyroid ultrasound but also creating artificial intelligence that integrates thyroid ultrasound images and clinical data of thyroid cancer, which is used to determine the diagnosis of thyroid cancer, and can also accurately predict postoperative survival period of thyroid cancer patients. It aims to provide new research directions for scientific researchers, and bring more personalized treatment plans for doctors and patients through the continuous progress of artificial intelligence technology, treatment strategies, and improve patients’ satisfaction and quality of life.
